# Navigating patient bias and mistreatment: a cross-sectional study of a workshop and coaching series for internal medicine residents

**DOI:** 10.1186/s12909-025-08047-0

**Published:** 2025-10-21

**Authors:** Julie Nguyen, Melisa Celaya, Donna Holland, Cheryl O’Malley, Emily Mallin

**Affiliations:** 1 Department of Internal Medicine, University of Arizona College of Medicine – Phoenix, Phoenix, AZ USA; 2 Banner – University Medical Center Phoenix, Phoenix, AZ USA

**Keywords:** Patient bias, Trainee mistreatment, Professional-Patient relations, Clinician wellness, Medical education, Coaching

## Abstract

**Background:**

Physicians are often challenged with navigating patient bias and mistreatment, contributing to burnout and decreasing psychological safety and trust. We sought to determine the effectiveness of a resident workshop series supported by faculty coaches on the ability to navigate mistreatment by patients.

**Methods:**

We implemented an educational program and coaching series in our internal medicine (IM) residency program at an academic teaching hospital. The participants included 48 PGY2 and PGY3 IM residents and 10 faculty coaches. We created a large-group four-part workshop series for residents over a 10-month period, a small-group faculty coaching skills development series, and small-group resident coaching sessions by faculty. Residents were surveyed before and after the intervention evaluating instances of and response to mistreatment from patients.

**Results:**

Forty-eight PGY2 and PGY3 IM residents (100% of each class) completed the curriculum, 42 (87.5%) of whom completed the presurvey and 33 (68.8%) of whom completed the postsurvey. At baseline, 34/42 residents (81.0%) reported experiencing and 39/42 (92.9%) witnessed some form of verbal mistreatment. The postsurvey results demonstrated increased confidence in responding to mistreatment, with a response of “Often” for direct verbal comments increasing from 14.7% preworkshop to 30.0% postworkshop (*p* < 0.0001 for trend). They also reported an increase in the willingness of upstanders engaging against mistreatment when witnessed, with the “Maybe” category changing from 28.2% to 11.8% and the “Always” category changing from 23.1% to 41.2% (*p* = 0.0023 for trend).

**Conclusions:**

Our intervention increased confidence in responding to mistreatment experienced by residents. The program, incorporating skill-building along with coaching, is a novel approach to empowering both residents and faculty to address various forms of patient mistreatment. The ability to effectively navigate mistreatment may positively impact psychological safety and build trust.

## Introduction

Resident physicians experience bias and mistreatment by some patients and their family members, which can create additional challenges in a learning and working environment [1–3]. Mistreatment is broadly defined [4] and will be recognized in this paper as any behavior that shows disrespect for the dignity of others, including (but not limited to) discrimination on the basis of race, gender, and religion [5]. Some specific forms of mistreatment by patients toward their physician include explicit refusal of care, questioning the clinician’s role, nonverbal disrespect, jokes or stereotypes, assertive inquiry into the clinician’s background, or contextually inappropriate comments [6]. Several studies have reported that practicing physicians and trainees receive targeted biased comments or behaviors from patients [7–9]. 

Intimidation, harassment, and discrimination of physicians are associated with multiple negative outcomes, including increased burnout, feelings of inadequacy, compassion fatigue, and suicidality [10–13]. Bystanders have also reported moral distress and uncertainty in how to respond when observing patient mistreatment against other physicians [6]. As a consequence of physician burnout and decreased physician wellness, there may be a negative impact on patient care, such as decreased work productivity, medical errors, low job satisfaction, and poor quality of patient care [14]. The culmination of these effects also negatively impacts medical institutions by decreasing both patient and physician trust in institutional support, leading to possible social and financial backlash [15–17]. 

Despite the high prevalence and awareness of patient bias and mistreatment, there is a lack of training and curricular interventions on how to address this problem [13, 18, 19]. Recent published curricula include workshop series [20–27] or forum theater [28, 29], while others reviewed overarching strategies when designing a general framework [30–32]. The training of bystanders, or witnesses to mistreatment of their fellow physician colleagues, is even less robust. The best practices for the education and empowerment of trainees, as well as clear outcomes, are current topics of research.

The use of coaching in medical education has become increasingly common as a tool to help trainees be their best selves. Coaching has been used in a variety of educational settings to improve various domains, such as technical skills, well-being, resilience, and patient safety [33]. The inclusion of coaching techniques in training to respond to and recover from events of mistreatment has great potential to augment this work. To our knowledge, standardized coaching tools have not been used in curricula for medical trainees in navigating mistreatment.

As reported in national data, internal medicine (IM) residents at our institution have reported an increased frequency of targeted patient bias, most commonly in terms of sex, race, ethnicity, and religion [5]. Our residents expressed discomfort in knowing whether or how to respond to such events and what reporting mechanisms exist. Furthermore, faculty members expressed a lack of confidence in effectively coaching residents through these difficult experiences.

We created a longitudinal large-group educational workshop and small-group coaching program to arm IM residents with knowledge and tools to help recognize, appropriately address and recover from incidents of verbal misconduct, both as potential target and as witness. The objectives of this study were to evaluate the impact of the program on: (1) resident awareness of mistreatment; (2) confidence in responding directly or as upstanders; and (3) awareness of and barriers to reporting of mistreatment.

## Methods

All 48 PGY2 and PGY3 residents (24 per class) in our internal medicine residency program participated in the workshop series. The workshops were embedded into an established longitudinal didactic experience not available in the first year of residency. Resident demographics are detailed in Table 1. Ten early- to mid-career faculty members from the divisions of hospital medicine or general internal medicine were recruited via email to voluntarily participate as coaches based on their demonstrated strengths in mentorship, resident support, empathy, curiosity, emotional intelligence, and a clear interest in coaching, though none had previous formal coaching training. The educational program was conducted from July 2021 through May 2022. The program included a large-group workshop series for residents, a coaching skills development series for faculty, and a small group coaching series following each workshop, during which residents and faculty debriefed and applied new skills.

**Table 1:** Resident demographics

Demographics	PRE (*n* = 42)	POST (*n* = 33)
	*N* (%)	*N* (%)
Gender		
Male	21 (50.0)	8 (33.3)
Female	17 (40.5)	8 (33.3)
Undeclared/Missing	4 (9.)	8 (33.3)
Year of Residency		
PGY2	22 (52.4)	13 (39.4)
PGY3	20 (47.6)	20 (60.6)
Ethnicity = Latino	2 (4.8)	1 (3.0)
Race		
American Indian/Alaskan Native	0	0
Asian	16 (38.1)	4 (12.1)
Black/African American	0	0
Native Hawaiian/Pacific Islander	0	0
White	18 (42.9)	10 (30.3)
Other	2 (4.8)	0
Undeclared*/Missing	6 (14.3)	19 (57.6)

*prefer not to say

The resident workshop series included four sessions with themes and learning objectives: (1) addressing the types and impact of mistreatment; (2) mitigating barriers to action and training bystanders to become “upstanders” by employing intervening strategies against a specific aggression [32, 34]; (3) promoting recovery from mistreatment; and (4) understanding the formal and informal systems in place for reporting events and creating psychological safety. Workshop speakers included a professional executive coaching team along with various experts in the topics of medical education, psychological safety and the clinical learning environment, wellness, and diversity, equity, and inclusion. Each workshop incorporated activities and implemented coaching tools for residents to reflect upon and practice between sessions. A complete list of workshop themes, objectives, and activities along with coaching tools incorporated into the program are listed in Table 2. Workshop scheduling was embedded into resident didactic blocks that were already in existence and accounted for differences in resident call schedules, ensuring full participation.

**Table 2:** Resident workshop plan with associated themes, coaching tools, session goals, and activities with learning outcomes

Workshop Session #	Workshop Theme	Coaching Tools Utilized	Session Goals	Workshop Activities	Learning Outcomes
1	Identification and impact of mistreatment	-Active listening -Behavior-impact statements -Reframing -Curiosity -Boundary setting	-Create shared understanding of disruptive patient behaviors -Understand possible causes -Identify potential impact on patient care, the team, and individuals	-Group share of examples of mistreatment -Self-reflection exercise -Goal-setting	-Awareness of mistreatment -Perceived impact on care and team dynamics
2	When and how to address mistreatment by patients	-Response frameworks -Accountability -Goal-setting	-Increase self and situational awareness -Clarify goals of engagement -Describe 5D framework to respond to incidents of mistreatment	-Group share of situations in which concepts and tools were utilized -Self-reflection exercise -Accountability and goal-setting	-Direct response to mistreatment -Barriers to action
3	Evolving from bystander to upstander	-Role-play -Accountability -Peer reflection	-Define upstander -Recognize effect of preparation on positive outcome -Apply framework to respond to incidents of mistreatment	-Group share of situations in which concepts and tools were utilized -Small-group role play -Accountability and goal-setting	-Upstander behavior -Willingness to intervene
4	Recovery, self-care, and team wellbeing	-Debrief strategies -Psychological safety tools -Mindfulness	-Outline personal and institutional resources -Delineate features of an effective debrief -Apply strategies to increase psychological safety in teams	-Group share of situations in which concepts and tools were utilized -Formula for sustainability activity -Accountability and goal-setting	-Recovery -Reporting systems -Psychological Safety

Faculty facilitators received both the workshop material and additional coaching training in advance of each session to strengthen their ability to support and coach residents. The faculty facilitators participated in a four-session series led by the executive coaching team. The coaching skills and tools taught during those sessions included techniques for active listening, the use of powerful questions, and goal setting (Table 2). As new skills developed, the faculty facilitators led small-group resident coaching sessions (4–5 residents per facilitator) soon after each workshop. These groups incorporated faculty- and peer-to-peer coaching, reflection, mindfulness, emotional intelligence, and communication skills. Groups debriefed workshops and using real examples from residents, applied coaching skills to prepare residents for potential future events and supported recovery from past events. Identical pre- and post-surveys were distributed to IM residents via QualtricsXM™ (Qualtrics, Provo, UT) from June 2021 to June 2022. The results were de-identified and accessible only by project leadership.

Resident and faculty compositions are reported via descriptive statistics of mode/median for central tendencies and frequencies for variability. Pre- and post-surveys of residents consisted of Likert-type scale questions, where residents indicated their level of agreement with statements on a 5-point scale ranging from “never” to “always.” Categorical variables were summarized by frequency and proportion and ordinal scores by median with interquartile range (IQR) and by mean with standard deviation (SD). Responses to survey questions were compared between the two time points (pre and post) via the bootstrap approach, with resampling performed at the study participant level and with 100,000 bootstrap samples for each test. The test statistic for any binary variable was the difference in proportions between the two time points, and that for any ordinal score was the difference in medians. The statistical software environment R (version 4.3.2) was used to implement the bootstrap test [35]. 

Our institutional event reporting system, called Converge Platform^®^, is primarily used for patient safety events, but can be used for any type of event. The institutional Office of Patient Safety queried the total number of reports from IM residents regarding mistreatment from July 2021 through June 2022 to see whether residents used this reporting mechanism for mistreatment at any point before, during or after the educational program.

## Results

Among the IM residents who completed the curriculum, 42/48 (87.5%) responded to the presurvey, and 33/48 (68.8%) responded to the postsurvey. The baseline results demonstrated that mistreatment was common. A total of 34/42 (81.0%) residents indicated personally experiencing mistreatment with unwanted behaviors (words or actions), and 39/42 (92.9%) reported witnessing some form of verbal mistreatment toward another member of the health care team. Of the 34 residents who reported personally experiencing mistreatment, 31 (91.2%) were identified as microaggressions, 21 (61.8%) were direct blatant comments, 11 (32.4%) were verbal threats of intimidation or violence, 11 (32.4%) were unwanted touching, and 2 (5.9%) reported other types of mistreatment.

**Fig. 1:**
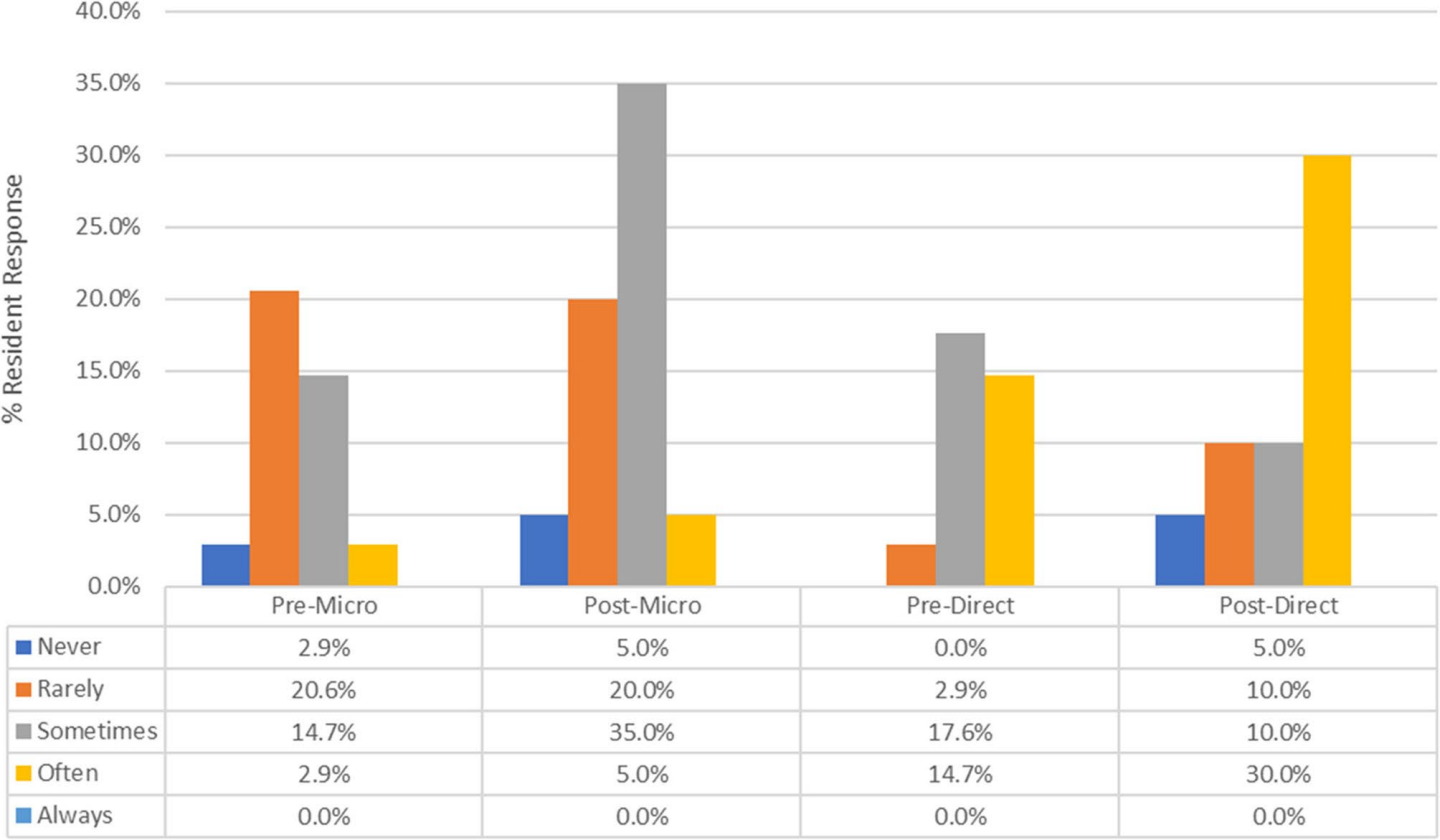
Direct response by residents to personal verbal mistreatment through microaggressions and direct verbal comments before and after the workshop (*p* < 0.0001)

**Fig. 2:**
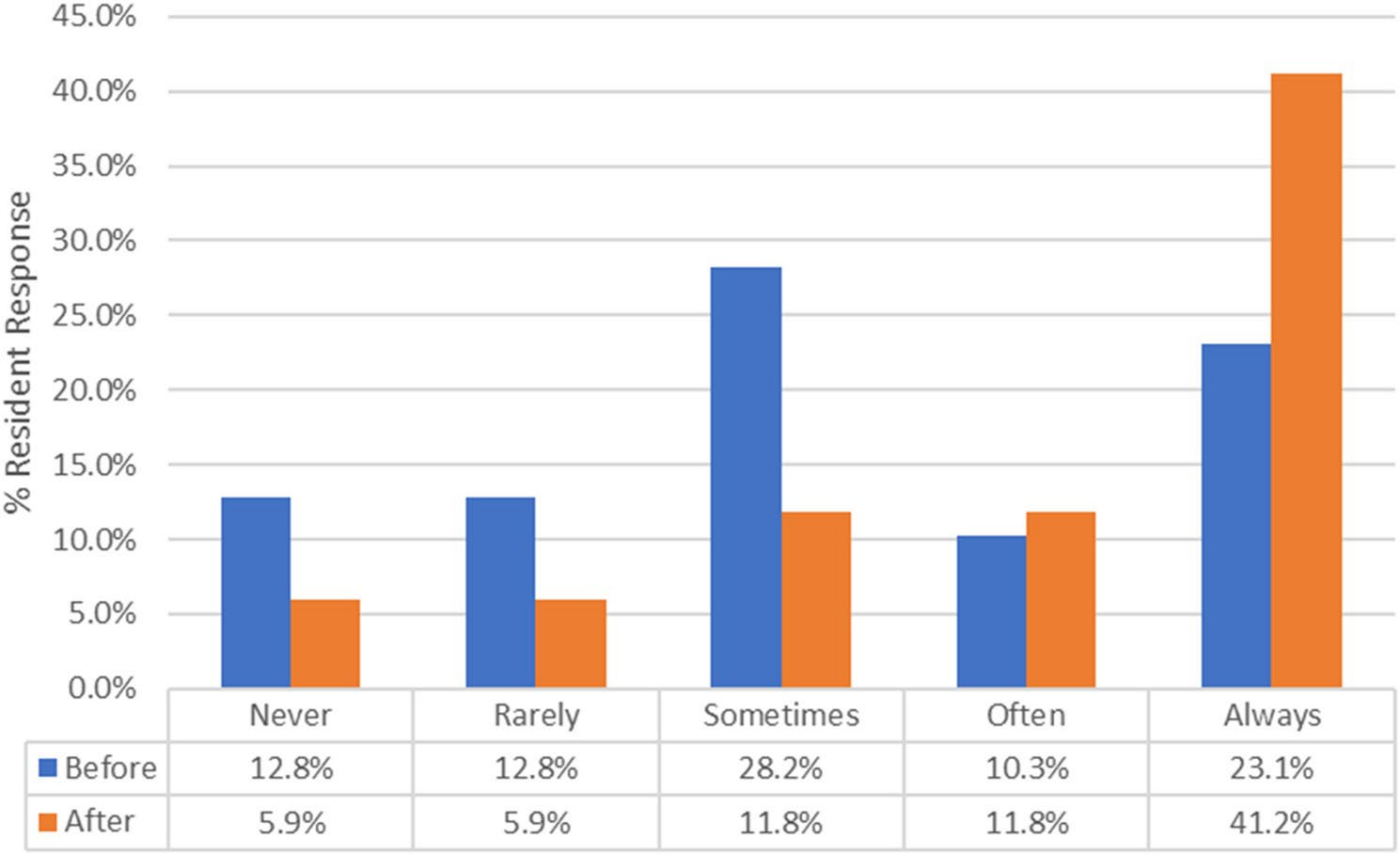
Bystander intervention to witnessed verbal mistreatment (*p* = 0.0023)

As depicted in Fig. 1, there was a significant increase in residents’ confidence in responding to personal verbal mistreatment through direct verbal comments before and after the workshop. For example, a response of “Often” for direct verbal comments changed from 14.7% preworkshop to 30.0% postworkshop (*p* < 0.0001 for trend). Similarly, Fig. 2 shows a significant increase in the willingness of upstanders to engage during witnessed incidents of mistreatment of other trainees. The most prominent changes were observed in the “Maybe” and “Always” categories, which changed from 28.2% to 11.8% (Maybe) and 23.1% to 41.2% (Always) (*p* = 0.0023 for trend), indicating that the residents were more willing to engage when witnessing verbal mistreatment after attending the workshop. The median frequency of direct response increased after workshop participation from 2.0 (rarely) to 4.0 (sometimes) for microaggression and from 4.0 (sometimes) to 5.0 (often) for direct comments and verbal threats. Similarly, the median frequency of direct response as an upstander on behalf of a peer increased from 4.0 (sometimes) to 6.0 (always).

We also measured verbal and written reporting of mistreatment by residents. A total of 9/48 (18.8%) residents verbally reported mistreatment in the presurvey to a colleague (7/9; 77.8%), senior or chief resident (5/9; 55.6%), faculty (6/9; 66.7%), program director (1/9; 11.1%), and other (2/9; 22.2%). There were no institutional electronic reports of mistreatment submitted by those who self-identify as residents in our institutional reporting system at any point in the year before or after the education program was initiated. Prior to the intervention, nearly half 19/42 (45.2%) of the residents were unaware of the process of submitting a written report of mistreatment, which improved to 3/33 (9.1%) after the workshop. Other barriers to reporting included the following: the incident did not seem important enough to report 13/42 (31.0%), the issue was resolved personally by the resident 14/42 (33.3%), the resident did not think the reporting would have changed anything 12/42 (28.6%), and fear of reprisal 6/42 (14.3%).

## Discussion

Our results demonstrate that the implementation of an educational program that includes skills workshops and coaching positively impacted the awareness and confidence of residents in navigating mistreatment by patients. Residents reported an increase in confidence in responding directly to both witnessed microaggressions and direct comments after they progressed through the program. There was also a significant increase in willingness to respond as an upstander to witnessed verbal comments toward others. Awareness of and instruction in our institutional system for reporting events increased as a result of the program, however, remained unused by residents for this purpose. It is not surprising that residents prefer to report mistreatment verbally, if at all, and to peers rather than leadership.

Some limitations exist in our study. Our single-program study has a small sample size, and the results may not be generalizable to other training programs or institutions. Because of the small sample size and the ordinal nature of the survey data, the bootstrap resampling method of analysis allowed us to avoid making assumptions about normal data distribution. Additionally, our intervention spanned an academic year, designed to be embedded in available didactic space to optimize repetition and application of material over time. The intended benefits of four workshops and four coaching sessions over time include reinforcement of learning and application of concepts and tools, increased psychological safety given the sensitive nature of the content, development of coaching relationships with faculty, and demonstration of the residency program’s commitment to supporting our residents facing these undesired situations. It is possible that this format may not be feasible for other institutions.

Because survey data came from resident self-report, there is no way to measure prevalence. Our institutional reporting system allows submissions for any type of patient-related events, and the submitter can self-identify with varying levels of anonymity, but other institutions may have different policies and procedures regarding a system-level tool. Regardless, this tool was not useful as a measure of resident mistreatment at our institution. Ultimately, we found that awareness of the process for formal reporting of these types of incidents was increased, however we are unable to draw any additional conclusions about ongoing barriers to institutional reporting.

The survey was designed to measure the overall impact of the program, as the use of coaching concepts and application of tools were key components of all aspects of the program and unable to be assessed separately. This limited our ability to measure the impact of the small-group coaching component separately. Although we did not perform formal psychometric testing to validate the questionnaire and confirm the reliability of the survey questions, the survey content and design were created by medical education faculty with expertise in trainee mistreatment, coaching and survey design. While the focus of our study was on impact on resident attitudes and behaviors, we solicited informal feedback from faculty coaches, which was generally positive.

Direct assessment of residents’ skills and behaviors in clinical practice after the intervention were not possible due to the unpredictable frequency of such events and potential impact of Hawthorne effect on patients and residents being observed. We also could not assess long-term retention of skills, attitudes or behaviors, as half of the participants received the final education and coaching sessions just prior to graduation. As in any study using self-reported data, our measured outcomes may not fully reflect actual behavior and could be influenced by participants’ desire to provide socially acceptable responses.

## Conclusion

Our residents were more aware of mistreatment by patients and the processes for reporting and indicated more confidence in responding to unwanted comments after the intervention. Overall, our study adds to the growing body of literature in support of interactive resident educational sessions to improve navigation of mistreatment and introduces coaching as an innovative complement to support residents in their approach to addressing mistreatment as target and as an upstander.

## Data Availability

The datasets generated and/or analyzed during the current study are not publicly available to preserve research participant anonymity but are available from the corresponding author upon reasonable request. Data are located in controlled access data storage at The University of Arizona.
